# Have advances in medical therapy for ulcerative colitis impacted surgical treatment?

**DOI:** 10.1002/ags3.12626

**Published:** 2022-10-11

**Authors:** Ryuichi Kuwahara, Hiroki Ikeuchi, Yuki Horio, Tomohiro Minagawa, Kurando Kusunoki, Motoi Uchino

**Affiliations:** ^1^ Division of Inflammatory Bowel Disease Surgery, Department of Gastroenterological Surgery Hyogo Medical University Nishinomiya Japan

**Keywords:** biological agent, medical treatment, prognosis, surgical treatment, ulcerative colitis

## Abstract

**Aim:**

The purpose of this study was to examine how the clinical characteristics, indications for surgery, and postoperative course of ulcerative colitis (UC) surgical patients changed before and after the introduction of biological agents.

**Methods:**

Patients who underwent surgery for UC at Hyogo Medical University between 2000 and 2019 were included in the study; those who underwent surgery between 2000 and 2009 were included in the early group (n = 864), and those who underwent surgery between 2010 and 2019 were included in the late group (n = 834); each study factor was retrospectively compared.

**Results:**

The mean ages at surgery (y) were 39.7 ± 15.1 y in the early group and 46.7 ± 17.8 y in the late group (*P < .*01). Antitumor necrosis factor agents were used (%) in 2 (0.2) patients in the early group and 317 (38.0) patients in the late group (*P < .*01). The number of cancer or dysplasia patients for whom surgery was indicated was significantly higher in the late group (11%/26%, *P = .*01). With regard to surgery in elderly individuals, the number of elderly patients (65 y and older) undergoing surgery was significantly higher in the late group (8.0%/18.6%, *P < .*01). For emergency surgery, the mortality rates were 16.7% (2/12) in the early surgery group and 15.7% (8/51) in the late surgery group (*P = .*61).

**Conclusion:**

The characteristics of UC patients requiring surgery in Japan have changed. The distribution of surgical indications changed, and the number of patients with cancer and dysplasia requiring surgery increased. The prognosis of elderly patients who underwent emergency surgery was poor.

## Graphical abstract

The characteristics of UC patients requiring surgery in Japan have changed. The number of patients with cancer and dysplasia requiring surgery increased. The prognosis of older patients who underwent emergency surgery was poor.

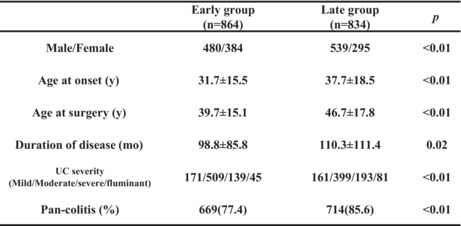

## 1 INTRODUCTION

The number of Japanese patients with ulcerative colitis (UC) has steadily increased, and is estimated to have exceeded 200 000.^1^ In Japan, medical treatment for UC began to advance in approximately 2000. Subsequently, the introduction of tacrolimus, which was covered by insurance in 2009, and infliximab, which was covered by insurance in 2010, began to have a significant impact on patients requiring surgery. Since then, numerous new agents have been introduced, and medical treatment options for UC have become increasingly available (Table 1). The goal of surgery for UC is to improve patients' quality of life and to save their lives. In recent years, the decision for surgical treatment has become more difficult due to the increase in medical treatment options. While surgery can be avoided in an increasing number of patients, emergency surgery is still needed. Leeds et al^2^ reported that prolonged preoperative medical treatment is a risk factor for postoperative complications, so caution should be exercised. In addition, the prolonged duration of UC compared to previous years may have led to an increase in inflammatory carcinogenesis in patients.

**TABLE 1:** Medical treatment and insurance coverage for ulcerative colitis in Japan

Year	New remedies and treatments
2000	Granulocyte and monocyte adsorptive apheresis (GMA)
2001	Leukocytapheresis (LCAP)
2006	Azathioprine
2009	Tacrolimus
2010	Infliximab (IFX)
2013	Adalimumab (ADA)
2017	Golimumab, Budesonide
2018	Vedolizumab, Tofacitinib

Thus, advances in medical therapy for UC may significantly impact the number of UC patients requiring surgery. The purpose of this study was to examine how the clinical characteristics, indications for surgery, and postoperative course in patients requiring surgery changed before and after the introduction of biological agents.

## 2 MATERIALS AND METHODS

### 2.1 Patients and data collection

Patients who underwent surgery for UC between 2000 and 2019 at Hyogo Medical University were included in the study. Patients who underwent surgery between 2000 and 2009 were defined as the early group (n = 864), those who underwent surgery between 2010 and 2019 were defined as the late group (n = 834), and each study factor (patient characteristics, duration of disease, preoperative medical treatments, surgical indications, death in the early postoperative period, surgery for elderly individuals) was compared retrospectively.

### 2.2 Definitions

The diagnosis of UC, the type of disease, and the phase of colitis activity were confirmed using the definitions developed by the Research Group for Intractable Inflammatory Bowel Disease. UC severity is graded as “mild” when (1) the frequency of defecation is 4 times/d or less, (2) bloody stools are rarely present, and (3) systemic symptoms such as fever, palpitations, and anemia are absent; UC is graded as “severe” when (a) the frequency of defecation is 6 times/d or more, (b) bloody stool is present, and (c) systemic symptoms such as fever, palpitations, and anemia are present, and “Fulminant” was defined as meeting all of the following five criteria. (i) met the criteria for severe UC, (ii) continuous bloody diarrhea of 15 or more episodes/d, (iii) persistent fever of 38°C or higher, (iv) white blood cell count of 10 000/mm3 or higher, and (v) severe abdominal pain.^3^ The indications for surgery were classified into the following three categories: refractory, cancer/dysplasia, and severe/fulminant.

Elderly patients were defined as those aged 65 y or older. Late‐onset UC was defined as patients with UC diagnosed at age 60 y or older. Surgery for UC was defined as an initial colorectal resection for UC, but ileostomy alone was excluded. Postoperative complications were determined to be those that occurred within the first month after surgery. Postoperative complications were defined as complications of grade 2 or higher according to the Clavien–Dindo classification.^4^ Mortality was defined as death within 30 d directly related to the surgical procedure. Surgery was defined as “elective” if the decision to operate for UC was made prior to admission to the hospital. In contrast, the decision to perform an “emergency” colectomy was made during or after admission for acute complications or for UC that was refractory to in‐hospital intensive medical management.

### 2.3 Statistical analysis

All statistical analyses were carried out using JMP vr. 15 (SAS Institute, Inc., Cary, NC, USA). The characteristics and surgical variables were compared with the Pearson χ^2^ test, Student's *t* test, or the Mann–Whitney *U* test. Statistical significance was set as a *P* value less than .05.

### 2.4 Ethical considerations

All study protocols were approved by the Institutional Review Board of the Hyogo Medical University (No. 3999).

## 3 RESULTS

### 3.1 Patient characteristics

The patients' clinical characteristics are shown in Table 2. The sex (male/female) ratios were 480/384 patients in the early group and 539/295 patients in the late group. There were significantly more males in the late group than in the early group (*P <*.01). The ages at onset (y) were 31.7 ± 15.5 y in the early group and 37.7 ± 18.5 y in the late group (*P <*.01). The ages at surgery (y) were 39.7 ± 15.1 y in the early group and 46.7 ± 17.8 y in the late group (*P <*.01). The duration of disease (months) was 98.8 ± 85.8 in the early group and 110.3 ± 111.4 in the late group (*P =* .02). The severity of UC (mild/moderate/severe) was 171/509/184 patients in the early‐stage group and 161/399/274 patients in the late‐stage group, with more severe cases in the late‐stage group (*P <*.01).

**TABLE 2:** Patient characteristics

	Early group (n = 864)	Late group (n = 834)	*P*
Male/female	480/384	539/295	<.01
Age at onset (y)	31.7 ± 15.5	37.7 ± 18.5	<.01
Age at surgery(y)	39.7 ± 15.1	46.7 ± 17.8	<.01
Duration of disease (mo)	98.8 ± 85.8	110.3 ± 111.4	.02
UC severity (Mild/moderate/severe/fulminant)	171/509/139/45	161/399/193/81	<.01
Pan‐colitis (%)	669 (77.4)	714 (85.6)	<.01

The prevalence of the pancolitis type (%) was 669 (77.4) in the early group and 714 (85.6) in the late group (*P <*.01).

### 3.2 Preoperative medical treatments

The preoperative medical treatments are shown in Table 3. The total steroid dose (mg) was 13 938.2 ± 15 020.8 in the early group and 8235.9 ± 15 085.9 in the late group (*P <*.01). The daily steroid doses (mg) were 22.9 ± 19.9 in the early group and 13.1 ± 19.2 in the late group (*P <*.01). Leukocyte apheresis (%) was used for 470 (54.3) patients in the early group and 357 (42.9) patients in the late group (*P <*.01). These three factors were significantly higher in the early group than in the late group.

**TABLE 3:** Preoperative medical treatment

	Early group (n = 864)	Late group (n = 834)	*p*
Steroid (total dose)	13 938.2 ± 15 020.8	8235.9 ± 15 085.9	<.01
Steroid (daily dose)	22.9 ± 19.9	13.1 ± 19.2	<.01
Immunosuppressive drugs (%)	180 (20.8)	482 (57.8)	<.01
Leukocytapheresis (%)	470 (54.3)	357 (42.9)	<.01
Anti‐TNFα agents (%)	2 (0.23)	317 (38.0)	<.01

Immunosuppressive drugs were used (%) in 180 (20.8) patients in the early group and 482 (57.8) patients in the late group (*P <*.01). Antitumor necrosis factor agents were used (%) in 2 (0.2) patients in the early group and 317 (38.0) patients in the late group (*P <*.01). These two factors were significantly higher in the late group than in the early group.

### 3.3 Surgical indications

Table 4 shows the indications for surgery. In the early group, 588 (68%) patients were refractory, 92 (11%) had cancer/dysplasia, and 184 (21%) had severe/fulminant disease. In the late group, 342 (41%) patients were refractory, 218 (26%) had cancer/dysplasia, and 274 (33%) had severe/fulminant disease. There were statistically significant differences in surgical indication (*P <*.01). The number of patients with cancer/dysplasia as an indication for surgery was higher in the late group than in the early group (early/late = 92/864 [10.6%]/218/834 [26.1%]). On the other hand, the number of patients with refractory indications for surgery was lower in the late group than in the early group (early/late = 588/864 [68.0%]/342/834 [41.0%]). Emergency surgery was higher in the late group, performed for 165 (19.1%) patients in the early group and 222 (26.6%) patients in the late group (*P <*.01). Details of cancer/dysplasia patients are shown in Table 5. Dysplasia was present in 27 (3.1%) patients in the early group and 56 (6.7%) patients in the late group, and cancer was present in 65 (7.5%) patients in the early group and 162 (19.4%) patients in the late group; both were significantly increased in the late group (*P <*.01).

**TABLE 4:** Surgical indication

	Early group (n = 864)	Late group (n = 834)		*P*
Refractory (%)	588 (68)	342 (41)	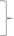	
Cancer/dysplasia (%)	92 (11)	218 (26)		<.01
Severe/fulminant (%)	184 (21)	274 (33)		

**TABLE 5:** Details of cancer/dysplasia cases

	Total (n = 1698)	Early group (n = 864)	Late group (n = 834)	*P*
Dysplasia (%)	83 (4.8)	27 (3.1)	56 (6.7)	<.01
Cancer (%)	227 (13.3)	65 (7.5)	162 (19.4)	<.01
Stage of cancer	0:74	00:27	00:47	.389
	I:63	I:15	I:46	
	II:52	II:11	II:40	
	III:19	III:5	III:14	
	IV:22	IV:7	IV:15	
UC onset to diagnosis of cancer (mo)	204.0 ± 115.9	188.7 ± 92.7	205.3 ± 124.3	.379

Regarding cancer stage (0/I/II/III/IV), there were 74/63/52/19/22 patients in the early group and 47/46/40/14/15 patients in the late group, respectively. There was no significant difference between the two groups (*P =* .389). The time from UC onset to cancer diagnosis was 188.7 ± 92.7 in the early group and 205.3 ± 124.3 in the late group (*P =* .379).

### 3.4 Death in the early postoperative period

Table 6 shows deaths in the early postoperative period. There were 11 deaths (1.3%) in the early group and 12 (1.4%) in the late group. There was no statistically significant difference between the two groups (*P =* .77). Deaths by surgical indication are as follows. Perioperative mortality in UC patients with refractory indications for surgery was 0.16% (1/608) in the early group and 0.25% (1/369) in the late group (*P =* 1.00). UC patients with cancer/dysplasia were 2.1% (2/92) in the early group and 0.9% (2/218) in the late group (*P =* .584). A total of 4.8% (8/164) of the patients in the early group and 4.1% (9/220) in the late group had severe/fulminant cases (*P =* .803). There was no significant difference in mortality due to surgical indications between the early and late groups. The most common causes of death were infectious complications such as pneumonia and sepsis in both groups. Other causes were cancer, hemorrhagic shock, intraabdominal hemorrhaging, and liver failure.

**TABLE 6:** Death in the early postoperative period

	Early group (n = 864)	Late group (n = 834)	*P*
Perioperative death (%)	11 (1.3)	12 (1.4)	.77
Pneumonia	5	5	
Sepsis	4	2	
Cancer	2	1	
Intraluminal/ remnant rectal bleeding	0	2	
Intra‐abdominal hemorrhage	0	1	
Liver failure	0	1	

### 3.5 Surgery for elderly individuals

Table 7 shows data on surgery in elderly individuals. Elderly patients requiring surgery included 69 (8.0%) patients in the early group and 155 (18.6%) in the late group. Patients requiring surgery with late‐onset UC (%) included 52 (6.0%) patients in the early group and 130 (15.6%) in the late group. This age group also had significantly higher surgery rates (*P <*.01).

**TABLE 7:** Surgery for the elderly

	Early group (n = 864)	Late group (n = 834)	*P*
Surgical cases for the elderly (%) (over 65 y old)	69 (8.0)	155 (18.6)	<.01
Surgical cases with elderly onset (%) (onset at age 60 or older)	52 (6.0)	130 (15.6)	<.01

Table 8 illustrates surgery and perioperative mortality for the elderly patients. The mortality rate of the elderly UC patients was higher in the emergency group than in the elective group (1.8% vs 16.7% *P =* .08) for both the early group and the late group (1.9% vs 15.7%, *P <*.01).

**TABLE 8:** Elderly surgery and perioperative mortality

	Elective surgery	Emergency surgery	*P*
Early group (n = 69)	1/57 (1.8)	2/12 (16.7)	.08
Late group (n = 155)	2/104 (1.9)	8/51 (15.7)	<.01

## 4 DISCUSSION

In recent years, remarkable progress has been made regarding medical therapy for UC. In this study, we showed a decrease in the use of steroids and cytopheresis and an increase in the use of immunomodulators and anti‐TNF. Danish and Canadian reports have increased the use of thiopurine and anti‐TNF and decreased the surgical rates of UC.^5^, ^6^ However, it is unclear how advances in medical therapy have affected UC patients undergoing surgical treatment and the indications for surgery.

In this report, the age at surgery was older, and the duration of illness was predominantly longer in the late group. Possible advances in medical treatment have increased the number of patients in whom surgery can be avoided. In this study, there was a significant increase in male patients in the late group. It has been reported that anti‐TNF is more effective in women,^7^, ^8^, ^9^ which may be one of the reasons for the increase in male surgical patients.

The age at which UC patients are operated on is increasing. With regard to the risk of UC patients becoming eligible for surgery, it is difficult to give an exact frequency because approximately half of the patients in this study were referred from other hospitals, and the total number of UC patients at other hospitals is not known. The cumulative risk of colorectal resection 10 y after diagnosis was estimated to be 28% in a Japanese report.^10^ However, a Chinese report in 2009 reported an incidence of 7.6% at 10 y after diagnosis.^11^

Refractory disease was the most common indication for surgery in both groups, but the number of cancer/dysplasia patients increased in the late group. Advances in medical treatment for UC have improved the rate of induction of remission and increased the number of patients in which surgery can be avoided. On the other hand, these patients have a longer disease duration, and the number of patients with cancer and dysplasia due to inflammation is increasing.^12^ Eaden et al reported that the cancer incidence rate of UC patients is 2.1% at 5 y, 8.5% at 10 y, and 17.8% at 20 y of disease duration.^13^ As medical therapy is expected to continue to advance, the number of patients with cancer/dysplasia will continue to increase.

Screening with colonoscopies is being promoted for the early detection of UC‐associated colorectal cancer (CRC) and dysplasia, which has been increasing in recent years.

The ECCO guidelines recommend screening for patients with UC with a disease duration of more than 6–8 y.^14^ In Japan, screening is also recommended for patients who have had UC for more than 8 y.^15^ Reductions in mortality due to colonoscopy screening has been reported in Japan and globally.^16^, ^17^ Screening will continue to be important for improving the prognosis of UC‐associated CRC. On the other hand, UC is difficult to detect even by specialists because of the inherent inflammation of the mucosa. The differential diagnosis of cancer, high‐grade dysplasia, and low‐grade dysplasia is not easy, and it is common for pathologists to disagree on the diagnosis. Some reports suggest that in potentially problematic patients, it is better to obtain the diagnosis from two independent pathologists who are skilled in diagnosing inflammatory bowel disease (IBD) tumors.^18^, ^19^ In Europe and the US, step biopsy is used, and in Japan target biopsy is used, but there are reports that the detection rate of neoplasias is the same for these two methods.^20^ Another problem is that there are no clear standards for screening intervals. Accumulation of data will be necessary to establish better screening schedules in the future.

Japan is an aging society with an average life expectancy of over 80 y in 2017,^21^ and more than 25% of the nation's population is at least 65 y old.^22^

Ten percent of newly enrolled patients with UC are over 65 y of age, and the number of patients with late‐onset UC is increasing.^23^ Furthermore, advances in medical treatment and an increased number of treatment options may have contributed to an increase in the number of elderly UC patients requiring surgery, increasing the number of patients in which surgery could be avoided. In fact, in the current study there was a significant increase in the number of UC surgical patients over 65 y of age in the late group and a significant increase in the number of UC surgical patients with an onset after the age of 60.

The number of elderly UC surgery patients is increasing due to the aging of UC patients and the increasing number of late‐onset UC patients. In particular, elderly patients with severe or fulminant UC often require emergency surgery. Elderly patients with UC have more comorbidities and are less likely to show symptoms, making therapeutic decisions difficult and potentially risky during surgery. In fact, we have reported that the perioperative mortality rate of elderly patients aged 60 y or older undergoing emergency UC surgery is higher than that of those undergoing elective surgery (0.88% vs 26.7% *P <*.01).^24^ In the present study, the perioperative mortality rate of elderly UC patients was higher in the emergency group than in the elective group. Therefore, we believe that emergency surgery should be avoided in elderly patients. We do not know the reason for the increased incidence of an elderly onset, but it is possible that the timing of surgery in elderly patients is more important and that the perioperative mortality rate is higher when emergency surgery is performed, which may lead to earlier identification for operation.

One of the most important factors is determining the limits of medical treatment is the timing of the decision for surgical treatment. This is very difficult to determine in elderly patients due to the presence of comorbidities and the fact that subjective symptoms may not be apparent. In recent y, the number of medical treatment options has increased, making the decision even more difficult. It is inevitable that elderly UC patients with low reserve capacity will have poor postoperative outcomes if their general condition worsens after prolonged medical treatment and emergency surgery is needed. In fact, it has been reported that the incidence of postoperative complications is higher in patients with longer preoperative hospital stays.^25^ In light of the above complications, it may be helpful to prioritize a literature review on the effects of medical therapy. Postoperative medical therapy is generally administered for 2 weeks in elderly UC patients. Early treatment planning may help prevent general deterioration before surgery in elderly patients. It may also be necessary to fully explain the high risk of emergency surgery during medical treatment.

Physicians and surgeons need to collaborate early on to determine a treatment plan, especially for elderly patients who have difficulty making decisions. When both physicians and surgeons explain the situation to the patient, the patient will be reassured and can move on to surgical treatment in a more appropriate time. In many areas, there are no surgeons who specialize in IBD. In these facilities, early consultation and transfer to a facility with an IBD center when the condition of elderly UC patients deteriorates (especially those with late‐onset UC) may lead to improved patient outcomes.

There are several limitations to this study. First, this was a single‐center, retrospective study. Second, Hyogo Medical University is one of the few institutions in Japan with an IBD center. Therefore, there is bias because more patients with severe IBD are seen at this center. As a result, the postoperative mortality rate may be higher. Second, most of the patients in this study period underwent open surgery, and the rate of laparoscopic surgery was low.

## 5 CONCLUSION

Since the advent of biologics, there were significantly more male patients requiring surgery, and the patients requiring surgery were elderly. The distribution of surgical indications changed, with an increase in the number of patients operated on for cancer/dysplasia. There was a significant increase in the number of emergency surgery patients. The prognosis for elderly patients undergoing emergency surgery was poor.

## DISCLOSURES

Conflicts of Interest: The authors have no conflicts of interest to declare.

Author Contributions: Conception and design: M Uchino, R Kuwahara, H Ikeuchi. Data acquisition: R Kuwahara, Y Horio, T Minagawa, K Kusunoki. Data analysis and interpretation: R Kuwahara, M Uchino. Drafting the article: R Kuwahara, H Ikeuchi. Critical revision of the article: R Kuwahara, M Uchino, H Ikeuchi, Y Horio, T Minagawa, K Kusunoki. Final approval of the article: R Kuwahara, M Uchino, H Ikeuchi, Y Horio, T Minagawa, K Kusunoki.

Approval of the Research Protocol: All study protocols were approved by the Institutional Review Board of Hyogo Medical University (No. 3999).

Informed Consent: This was a retrospective study, and consent for participation was waived.

Registry and the Registration No. of the study/Trial: Institutional Review Board of the Hyogo Medical University (No. 3999).

Animal Studies: N/A.
